# Secondary Metabolites and Antioxidant Activity against Moko Disease as a Defense Mechanism of *Musa* spp. from the Ecuadorian Coast Area

**DOI:** 10.3390/metabo14060307

**Published:** 2024-05-28

**Authors:** Raluca A. Mihai, Vanessa A. Terán-Maza, Karen A. Portilla-Benalcazar, Lissette E. Ramos-Guaytarilla, María J. Vizuete-Cabezas, Erly J. Melo-Heras, Nelson S. Cubi-Insuaste, Rodica D. Catana

**Affiliations:** 1 Army Scientific and Technological Research Center—CICTE, Department of Life Science and Agriculture, Universidad de Las Fuerzas Armadas—ESPE, Av. General Rumiñahui s/n y, Sangolqui 171103, Ecuador; vateran1@espe.edu.ec (V.A.T.-M.); kaportilla@espe.edu.ec (K.A.P.-B.); leramos4@espe.edu.ec (L.E.R.-G.); mjvizuete@espe.edu.ec (M.J.V.-C.); ejmelo@espe.edu.ec (E.J.M.-H.); nscubi@espe.edu.ec (N.S.C.-I.); 2 Developmental Biology Department, Institute of Biology Bucharest of Romanian Academy, 296 Splaiul Independenţei, 060031 Bucharest, Romania; rodica.catana@ibiol.ro

**Keywords:** flavones, kaempferol-7-O-neohesperidoside, LC-MS, phenolic content, *Ralstonia solanacearum* race (phylotype) 2

## Abstract

The *Musa* spp. represents the most commonly produced, transitioned, and consumed fruit around the globe, with several important applications in the biotechnology, pharmaceutical, and food industries. Moko disease is produced by *Ralstonia solanacearum*—a factor with a high impact on all crops in Ecuador, representing one of the biggest phytosanitary problems. Four of the most common varieties of *Musa* spp. were tested to identify the metabolic reaction of plants facing Moko disease. The phenolic and flavonoid content has been evaluated as a defense system, and the α-diphenyl-α-picrylhydrazyl free-radical-scavenging method (DPPH), free-radical-scavenging activity (ABTS), ferric-reducing antioxidant power (FRAP) assays, and liquid chromatography and mass spectrometry (LC-MS) have been adapted to analyze the active compounds with the antioxidant capacity necessary to counteract the pathogenic attack. Our results indicate that all the studied varieties of *Musa* spp. react in the same way, such that the diseased samples showed a higher accumulation of secondary metabolites with antioxidant capacity compared with the healthy ones, with high active compound synthesis identified during the appearance of Moko disease symptoms. More than 40 compounds and their derivatives (from kaempferol and quercetin glycosides) with protective roles demonstrate the implication of the *Musa* spp. defense system against *R. solanacearum* infection.

## 1. Introduction

The *Musa* spp. (Musaceae family) enjoys increased interest since bananas represent the world’s most commonly produced, traded, and consumed fruit, contributing to food security and incomes, and being in fourth place in importance in numerous countries [1]. Ecuador is the largest exporter globally, among Africa, Asia, and Latin America, which produce and sell bananas [2]. Besides its economic importance, the interest in banana plants grew due to their numerous uses in pharmacy, biotechnology, and food [3].

Among the factors that may affect banana manufacture, diseases caused by bacteria have serious effects [4]; *Ralstonia solanacearum* is one of the top 10 bacterial pathogens worldwide [5], characterized by a wide host range and wide geographic distribution, making it one of the most destructive crop pathogens in the world [6]. An alert was issued in February 2023 regarding Moko disease in Ecuador, reported in *Musa* plantations in more than 12 provinces [7].

Moko disease represents one of the phytosanitary problems with a great economic impact on all banana varieties and plantain crops, which affects over 70% of yield losses [6], as reported in a variety of studies regarding the treatment of this disease. Over time, some studies have been concerned about the Moko disease in *Musa* spp. In this way, molecular approaches like RAPD (Random Amplified polymorphic DNA) and rep-PCR-based fingerprinting (Repetitive sequence-based Polymerase Chain Reaction [8], AFLP molecular markers (Amplified Fragment Length Polymorphism) [9], and real-time PCR [10] were used to investigate the genetic relationship between *Ralstonia solanacearum* and *Musa* spp. diseases. Different strategies were tested to control/or eradicate the Moko disease: resident varieties [11,12,13,14], banana leachates [15], insects and beneficial microorganisms [16].

*R. solanacearum* invades the vascular tissues, causing the leaf’s death, which reduces the ability to produce fruit. Insects and/or contaminated farming equipment instead of mechanical and soil are the principal transmission vectors [17]. Recent genetic studies showed that bacterial strains can survive more than 25 years, affecting native flora, organic matter in soils, and crop plants in tropical and subtropic zones [18]. Even though there are some good practices (chlorine dioxide) and resistance inducers in banana plants [19] for farmers to reduce the spread of the disease agent, no cure is available for Moko.

Secondary metabolites are directly involved in plant defense; factors like genetic, ontogenic, morphogenetic, and environmental factors may affect their biosynthesis/accumulation [20]. Recent studies have pointed out that banana flavonoids can form complex compounds with extracellular proteins that can damage the cell membrane of bacteria, followed by the release of intracellular compounds [21]. Our study aimed to demonstrate that the antioxidant capacity resulting from the biosynthesis of secondary metabolites may act as a defense mechanism against the Moko attack in *Musa* spp. To achieve this aim, four of the most commonly used *Musa* spp. were investigated in our research, such as *Musa cavendish* Paxton [22]—the most widespread edible variety [23], *Musa paradisiaca* L.—a plantain used as a nutritional and therapeutic source [24], *Musa textilis* Née, an important source of fibers [25] and *Musa acuminata* Colla—the most frequent species [26].

## 2. Materials and Methods

### 2.1. Chemicals

To quantify the different secondary metabolites, the following chemicals (Sigma-Aldrich Chemical Co.—St. Louis, MO, USA) were used: 2.2—diphenyl—1—picrylhydrazyl, diammonium salt of 2.2—azinobis (3–ethylbenzothiazoline– 6-sulfonic acid), 2.4.6-tri(2-pyridyl)-triazine, iron (III) chloride hexahydrate and Folin–Ciocalteu reagent, 6-hydroxy-2.5.7.8-tetramethyl chroman-2-carboxylic acid, 3.4.5-trihydroxy benzoic acid, potassium persulfate, iron (III) sulfate heptahydrate, quercetin, and ethanol. All the reagents were purchased from commercial providers.

### 2.2. Sample Collection and Processing

This study was carried out with plants of the genus *Musa* spp., from farms located in Santo Domingo de los Tsáchilas province (0°05′01.6″ N, 79°26′32.8″ W), Canton La Concordia, km 38 road Santo Domingo—Quinindé. Healthy plants and plants with Moko disease symptomatology, at different stages of disease development, were collected. Each degree of infection (disease score) has been characterized and identified based on the following characteristics implemented by the Government of Kerala in 2014: Stage 1 (initial stage) symptoms are characterized by the yellowing of the central leaf, transitioning from yellow to green, weakening, and eventually breaking at the junction with the petiole. As the infection progresses, it leads to wilting and drying of young leaves, spreading to older ones. Older leaves then exhibit yellow bands with dark margins on their edges. In Stage 2 (intermediate stage), symptoms appear swiftly in the suckers, characterized by the progressive yellowing and wilting of older leaves. Additionally, small suckers exhibit a gradual death of the central leaf, which extends toward the outer leaves. In Stage 3 (advanced stage), symptoms in infected corms become evident through transverse cuts, revealing brown or black bands in the vascular bundles infected by the bacteria. Diseased pseudostems excrete bacterial exudate, while internally, vascular bundles exhibit a light to dark brown color due to blockage by extracellular polymeric substances. Plants without clusters display symptoms characterized by the grouping of vascular bundles near the pseudostem center, with significant blockages primarily observed in the petioles of the initially infected leaves, becoming less apparent toward the periphery. All the collected banana plants were in the vegetative stage, young plants of approximately 1 year old. The samples were transferred in a cooler to the university laboratory in Cantón Rumiñahui.

### 2.3. Active Ingredients Extraction

To determine the relationship between the antioxidant character and the phytochemical compounds involved in the defense of plants against Moko disease, in the first phase, the impurities were removed from the leaves by washing them with distilled water, and afterwards, the leaves were dried and ground (5 g) and placed in 25 mL of ethanol of HPLC (High Performance Liquid Chromatography) grade 99.5 (*v/v*), overnight, in the dark, at 4 °C.

### 2.4. Active Ingredients Determination

The Folin–Ciocalteu method was used to determine the phenolic concentration in the banana leaves [27]. Solutions from the ethanolic extracts (0.4 mL), Folin–Ciocalteu reagent (2 mL of 10% (*v*/*v*), and 7.5% Na_2_CO_3_ (1.6 mL) were incubated at room temperature for 30 min. The 765 nm absorbance was used to measure the concentrations. Gallic acid (0–250 mg/L concentration) was used for the calibration curve (y = 0.0112x + 0.1759, R^2^ = 0.9794).

A colorimetric method using aluminum chloride described by Pekal et al. [28] was used for the determination of the flavonoid content. Quercetin was used for the realization of the standard solution. The standard calibration curve was performed at a concentration between 0 and 1.5 mg L^−1^, obtaining the equation y = 1.4566x + 0.0265 with a correlation factor R^2^ = 0.9935. To the crude extracts of the plant sample (1 mL) was added 1.5 mL of the solvent, 100 µL of CH_3_COONa (1 M), 100 µL of AlCl_3_ (10% *v/v*), and distilled water (2.3 mL). The samples are covered for 40 min at room temperature. In the blank sample, the aluminum chloride was not placed. Finally, the samples were measured at 435 nm absorbance.

### 2.5. Antioxidant Capacity Determination

A protocol established by Sachett et al. (2021) [29] and modified by Thaweesang [27] was used for the determination of the antioxidant capacity through the 2,2-diphenyl-1-picrylhydrazyl (DPPH) radical. A stock solution of 1 µg L^−1^ was produced; 2 mL of stock was added to every laboratory test tube, including the 0.1 mL crude extracts sample. After the test tubes were left to incubate for 30 min at room temperature, the absorbance was measured at 517 nm and the radical scavenging activity was calculated using the following formula: control absorbance—sample absorbance/control absorbance ×100. The calibration curve (y = 158.07x − 1.6766, R^2^ = 0.9955) was produced using a Trolox solution (positive control) with a concentration between 0 and 0.625 mM.

The establishment of the antioxidant capacity by 2,2′-azino-bis(3-ethylbenzothiazoline-6-sulfonate) radical cation (ABTS^*+^) free radical scavenging (obtained from the reaction between ABTS (7 mM) and potassium persulfate) was based on a study by Kuskoski et al. [30]. After incubating the solution for 12–24 h, it was diluted in absolute ethanol until 0.7 ± 0.1 at 754 nm absorbance was obtained. The inhibitory capacity was determined after the reaction between the ABTS solution (2 mL) and sample (20 µL) for 7 min at room temperature and in darkness; absolute ethanol was used for the blank. The calibration curve was established between a 0 and 2.5 mM concentration, obtaining the following equation y = 31.995x + 3.9568 with a correlation factor R^2^ = 0.9697, using Trolox (like a positive control).

To determine the antioxidant capacity based on the Ferric ion Reducing Antioxidant Power (FRAP) test, a FRAP solution was prepared from acetate buffer 300 mM (100 mL) pH 3.6 + HCl (40 mM) + FeCl_3_ · 6 H_2_O (20 mM) in the proportion of 100: 10: 10 according to the method of Rajurkar et al. (2011) [30]. The absorbance of the mixture was read at 593 nm. The standard curve (y = 0.3654x − 0.0532, R^2^ = 0.9686) was performed by taking different concentrations of FeSO_4_ · 7H_2_O ranging from 0 to 5 mM. The solution consisting of sample (100 µL), distilled water (300 µL), and FRAP solution (3 mL) previously prepared was covered for half an hour at room temperature. The FRAP value (mmol Fe^2+^ g sample) was obtained by the following formula:

FRAP value = Sample absorbance − Control absorbance

### 2.6. LC-MS Determination

High-resolution LC-MS was used to determine the bioactive compounds in the samples of stem and leaves from *M. cavendish* at stage 1 of Moko disease. The plant extractions were established using a modified method based on Tohma et al. [31]. Ethanolic extracts were obtained from lyophilized samples represented by leaf and stem (1 g) and 80% ethanol (20 mL) maintained for 2 h at 30 °C and centrifuged for 10 min at 5000 rpm at 4 °C. Afterwards, the extracts were filtered and a rotary evaporator at 30 °C was used for ethanol removal. The samples were kept in well-covered plastic tubes until analysis was performed at −20 °C.

Identification and detection of different metabolites were performed by HPLC equipment (Vanquish Thermo Fisher Scientific (Waltham, MA, USA)) and Mass Detector (Ion Trap Thermo Fisher Scientific (Waltham, MA, USA)). The samples were eluted through an Accucore Vanquish 150 × 2.1 mm column at 35 °C and a 0.5 mL/min flow rate [32]. A 10 μL volume of 0.1% formic acid was used as a mobile phase to inject into the HPLC. MS scanning was used to identify the compounds from the samples based on the retention time comparison of each peak and the monitoring of the ion pairs in a standard solution [33]. The compounds were identified by comparing them with those of the standard compounds available in databases (PubChem, ChEBI, Metlin, HPLC, and the literature data) using the MZmine 2.53 software [34].

### 2.7. Statistical Test

The RStudio statistical program (1.3.8 version) was used. Significant differences were analyzed through a two-way ANOVA test (*p* < 0.05). All the experiments were realized in triplicate, with the values being presented as the mean ± SD. The correlation between the secondary metabolites analyzed and the antioxidant power was evaluated by Pearson’s correlation coefficient, while the correlation matrix was depicted as a scatter plot matrix.

## 3. Results

### 3.1. Active Ingredient Determination

**Figure 1:**
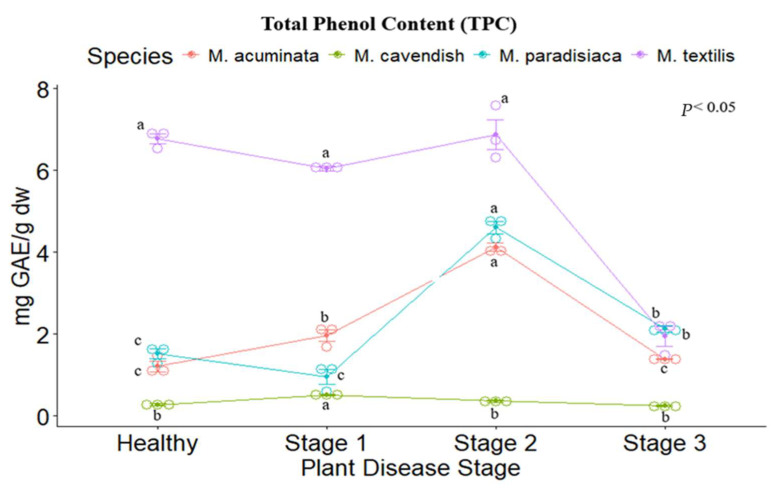
Total phenolic content of the tested *Musa* species. Legend: Stages 1, 2, and 3 represent the Moko disease stages. The plot is represented by the concentration in mg GAE/g dw on the *y*-axis, while the *x*-axis represents the stage for each plant species. Different letters denote significant differences.

The total phenolic content was evaluated for each disease stage per species. For *M. cavendish*, the highest phenolic content was registered in Stage I (0.507 ± 0.025 mg GAE g dw), Stage II (0.358 ± 0.052 mg GAE g dw), followed by healthy plants (0.27 ± 0.036 mg GAE g dw), while Stage III presented the lowest values. In contrast, for *M. acuminata, M. paradisiaca* and *M. textilis*, the highest phenolic content was registered in Stage II (4.114 ± 0.145 mg GAE g dw; 4.604 ± 0.215 mg GAE g dw, and 6.868 ± 0.526 mg GAE g dw, respectively), while the other stages presented a similar tendency. *M. textilis* showed the highest phenolic content of all the evaluated *Musa* spp. (Figure 1).

**Figure 2:**
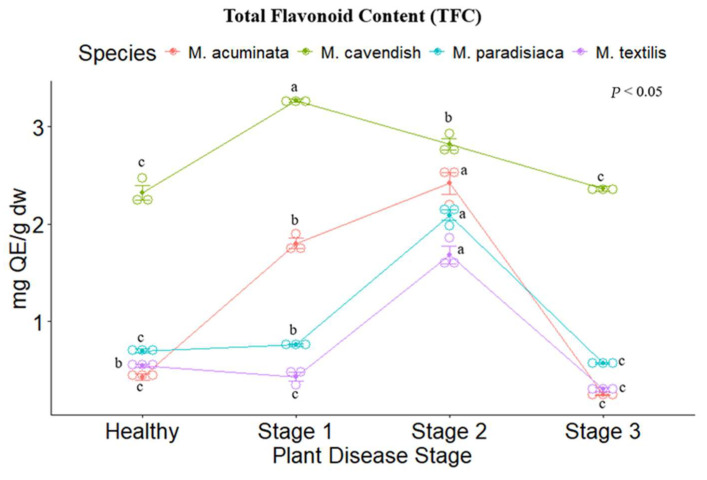
Total flavonoid content of the tested *Musa* species. Legend: Stages 1, 2 and 3 represent the Moko disease stages. The plot is represented by the concentration in mg QE/g dw on the *y*-axis, while the *x*-axis represents the stage for each plant species. Different letters denote significant differences.

*M. cavendish* showed the highest flavonoid concentration in contrast to the other species, with Stage I presenting the highest value (3.266 ± 0.0295 mg QE/g dw). The other *Musa* spp. show a similar trend to the phenolic content, as the highest value was reported for *M. acuminata, M. paradisiaca,* and *M. textilis*, appearing for Stage II (2.419 ± 0.197, 2.091 ± 0.0937, 1.682 ± 0.154, respectively), followed by the healthy stage, and Stages I and III with similar values. The lowest flavonoid content was reported for *M. textilis* (Figure 2).

### 3.2. Antioxidant Activity Determination

**Figure 3:**
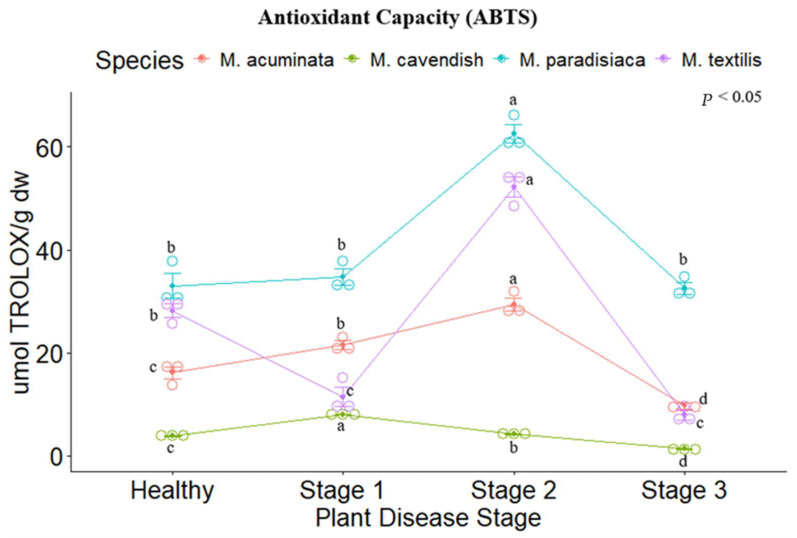
ABTS radical-scavenging assay of the tested *Musa* species. Legend: Stages 1, 2 and 3 represent the Moko disease stages. The plot is represented by the concentration in µmol Trolox/g dw on the *y*-axis, while the *x*-axis represents the stage for each plant species. Different letters denote significant differences.

**Figure 4:**
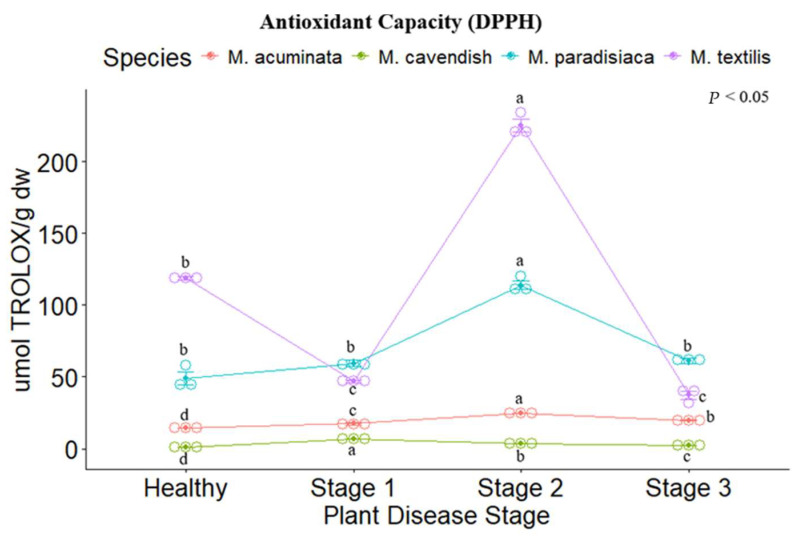
DPPH radical-scavenging assay of the tested *Musa* species. Legend: Stages 1, 2 and 3 represent the Moko disease stages. The plot is represented by the concentration in µmol Trolox/g dw on the *y*-axis, while the *x*-axis represents the stage for each plant species. Different letters denote significant differences.

**Figure 5:**
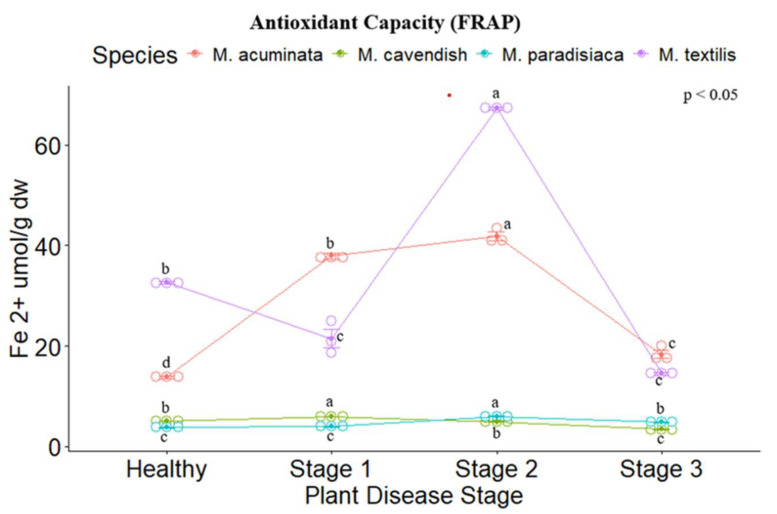
FRAP-reducing potential assay (µmol Fe ^2+^ g dw) of the tested *Musa* species. Legend: Stages 1, 2 and 3 represent the Moko disease stages. The plot is represented by the concentration in µmol Fe^2+^/g dw on the *y*-axis, while the *x*-axis represents the stage for each plant species. Different letters denote significant differences.

The antioxidant capacity of the four *Musa* spp. was evaluated through ABTS, DPPH, and FRAP assays. Significant differences were found between the disease stages for each species, following the same trend as their respective results concerning the secondary metabolites analyzed. For the ABTS and DPPH methods, *M. paradisiaca* (ABTS: 62.545 ± 2.524 µmol TEAC (Trolox Equivalent Antioxidant Capacity) g dw, DPPH: 113.997 ± 4.451 µmol TEAC g dw) and *M. textilis* (ABTS: 52.212 ± 2.742 µmol TEAC g dw, DPPH: 225.212 ± 6.708 µmol TEAC g dw) showed the highest values for Stage II. (Figure 3 and Figure 4), while for the FRAP assay. *M. acuminata* (41.803 ± 1.179 µmol Fe ^2+^ g dw) and *M. textilis* (67.24 ± 0.284 µmol Fe ^2+^ g dw) presented the highest values (Figure 5).

**Figure 6:**
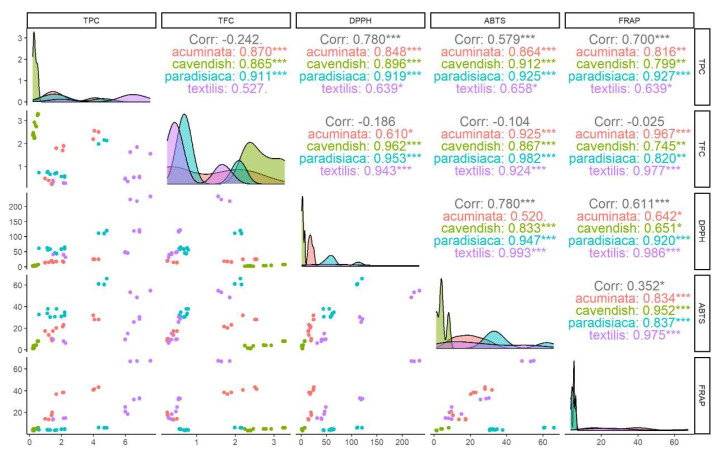
Correlation of flavonoids and total phenolic content with the ABTS, DPPH, and FRAP assays in the *Musa* species at different stages of *R. solanacearum* infection. Correlation values are expressed per species, classified by *** strong correlation, ** medium correlation, and * low correlation.

The correlation between the two analyzed secondary metabolites was raised for the four *Musa* spp. The contents were correlated with the three methods of antioxidant capacity evaluation used, with each plant showing a higher positive correspondence (R > 0.90) for the total phenolic content and flavonoid content. In contrast, for the phenolic content. *M. textilis* showed a lower correlation, albeit still positive with the ABTS (R = 0.64), DPPH (R = 0.66), and FRAP (R = 0.64) assays (Figure 6).

### 3.3. LC-MS Determination

**Figure A1:**
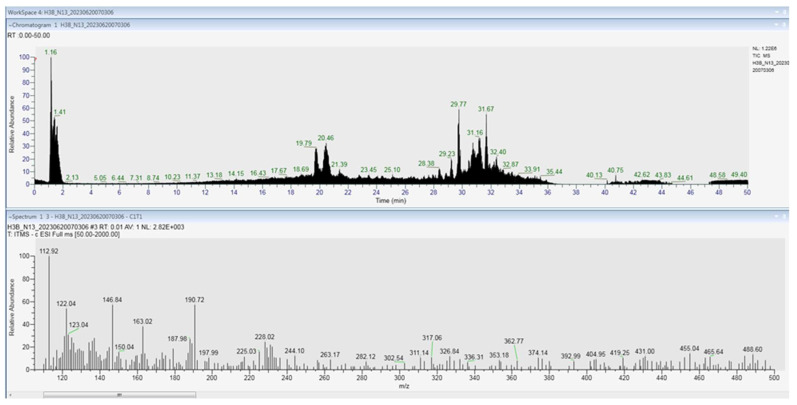
LC-MS chromatogram of the compounds identified in *Musa cavendish* leaf samples at Stage 1 of disease. The *y*-axis depicts the relative absorbance, while the *x*-axis represents the retention time (rt) in the upper chromatogram, and the identified molar mass is represented on the lower chromatogram for each sample.

**Figure A2:**
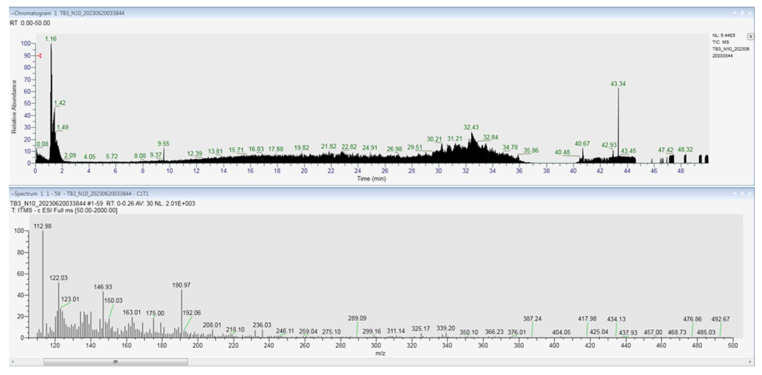
LC-MS chromatogram of the compounds identified in *Musa cavendish* stem samples at Stage 1 of the disease. The *y*-axis depicts the relative absorbance, while the *x*-axis represents the retention time (rt) in the upper chromatogram, and the identified molar mass is represented on the lower chromatogram for each sample.

LC-MS was used to determine the bioactive compounds in the samples of stem and leaves from *M. cavendish* at Stage 1 of Moko disease. The compounds in the extracts were identified based on their molecular mass and retention time. The phenolic extracts from the stem (34 identified compounds) and leaf (61 identified compounds) samples yielded similar HPLC profiles. However, differences were observed between both samples in terms of the identified phenolic compounds. The leaf and stem samples showed a similar content of phenol precursors such as shikimic acid, caffeoyl alcohol, coumaroyl, and derivatives of amino acids. Flavonoids were exclusively found in the leaf samples, with derivatives of quercetin (spiraeoside, rutin), kaempferol (astragalin, kaempferol-7-O-neohesperidoside), naringenin derivatives, and other flavonoid glycosides. Flavones such as isoorientin and scutellarin 4′-methyl ether had also been identified in the leaf samples. Citric acid, gibberellic acid, and terpenes (e.g., 7-O-methyl rosmanol) were identified mostly in the stem (Table 1, Figure A1 and Figure A2).

## 4. Discussion

Banana, as a key crop for food security, is considered the primary food for several developing countries [13]. However, plants from the genus *Musa* spp. are susceptible to some pathogens, including *R. solanacearum* race 2. The wilt disease is determined by bacterial accumulation, which blocks the vessels, stopping the sap flow [43]. Bacterial wilt lowers the yield in banana production [13]; therefore, treatment and management of this bacterial disease involve the achievement of advanced technologies together with research. The understanding of the interaction between *Musa* spp. and pathogens will allow for identifying new strategies to be used to control the diseases without damaging the environment. Physical barriers, vulnerability, and phytoalexin yielding are some of the recognized traits used by *Musa* plants as defense mechanisms against pathogens [44,45]. Although the crops are indeed the most affected, plants possess different mechanisms to protect themselves, such as the development of secondary metabolites to eliminate the pathogen from its system.

In our case, all four of the most commonly used *Musa* spp. investigated (*M. cavendish*, *M. paradisiaca*, *M. textilis,* and *M. acuminata*) showed a similar trend in front of infection with *R. solanacearum* race 2. The diseased samples exhibited a higher antioxidant activity compared to the healthy samples, and we attempted to show that this is attributed to the presence of secondary metabolites (phenolic compounds) through the LC-MS analysis to reinforce the idea that secondary metabolites and implicitly antioxidant activity represent a defense mechanism against the pathogen that causes Moko disease. Plants possess physical and chemical barriers involved in their defense; they have a strategy to help their survival when in contact with some biotic or abiotic stresses, implicating the synthesis of secondary metabolites, which is well known as part of the plant immune system. A strong emphasis is placed on antioxidant activity to increase plant protection against the pathogen. The synthesis of metabolites comes from the primary metabolism (glycolysis, Krebs cycle, or shikimate pathway) that depends on the degree of stress to which the plant is subjected and can trigger variance in the levels of secondary metabolites, some of them toxic when stored in plant cells [46]. Synthesized phenolic compounds like simple phenols, flavonols, dihydrochals, and cones phenolic acids are antibiotic compounds that generate a response against pathogens. Phytoalexins, synthesized de novo, manage to inhibit a diversity of microorganisms by their accumulation at the infected site [47].

The antioxidant capacity of plants is related to the defense of both types of antioxidants (enzymatic and non-enzymatic) to escape from the toxic effects of free radicals. Their genetic configuration confers a great capacity to synthesize secondary metabolites under biotic or abiotic stress. Some compounds act like substrates in enzyme-catalyzed detoxification reactions and have central and interrelated functions [48,49,50].

Likewise, there is a considerable difference between plant developmental stages. The vegetative stage of the samples shows a lower content of secondary metabolites and antioxidant activity. The bioactive compounds produced depend on the environmental conditions to generate an adequate impact on the development of the metabolic pathways associated with their biosynthesis [51]. In the vegetative state, plants use photosynthesis and carbon assimilation for growth, development, and defense, and when exposed to some stress, can alter the storage and synthesis of metabolites. These include the harvest time, exposure to factors such as light, temperature, osmotic potential, nutrition, growth regulators, biotic inducers, and fruit-ripening stage, among others [52].

Based on their elemental role in plant protection against different agents, phenolics are known as antioxidants [53]. Phenolic compounds exhibiting significant antioxidant compounds were identified in each *Musa* spp. The predominant polyphenols in the plant defense mechanism are flavonoids, which may be classified as flavonols, isoflavonols, flavones, flavanones, catechins, and anthocyanidins [54]. New research has shown the involvement of flavonoids in plant protection, playing a significant role in the neutralization of free radicals [55], especially in terms of the highly sensitive antimicrobial effect on pathogens, in which compounds such as naringenin, kaempferol, quercetin, and dihydroquercetin stand out [56]. Recent studies have described the antibacterial effect of phenolic compounds obtained from plantain leaves against some Gram-negative bacteria (*Escherichia coli*, *Staphylococcus*, *Pseudomonas)* species [57,58]. The bacterium *Ralstonia solanacearum* race 2 (Smith, 1896) is a Gram-negative bacillus with high genetic variability that affects the vascular system of the plant [59]. The phenolic compounds identified in the current study have shown antibacterial activity toward the infection since this strain is responsible for causing the Moko disease in *Musa* spp., whose direct effect is aggravated by the capacity of its causative agent to remain in the soil for a long time, disabling the immediate replanting of the affected lots [60], highlighting the importance of describing plant defense mechanisms to reach a better understanding of the infection. In our current study, these compounds and their derivatives have been identified, from kaempferol (astragalin, kaempferol-7-O-neohesperidoside) and quercetin glycosides (spiraeoside, rutin), as well as flavones like isoorientin and scutellarein 4′-methyl. Quercetin and its derivatives also play significant roles in plant protection from the effects of UV radiation and/or osmotic stress, in which glycosylated derivatives are involved in osmoregulation [61]. In addition, flavonoids are known for their antioxidant capacity by decreasing ROS levels by inhibiting prooxidant enzymes, cyclooxygenase, and lipoxygenase [62].

Furthermore, signaling molecules have been identified; products of oxidative processes’ ROS (7-methyl-rosmanol), supporting the idea of the loss of some acids (carnosic acid) with antibacterial activity against both bacteria types (Gram-positive and Gram-negative) causing the accumulation of oxidized derivatives under oxidative degradation by ROS [63,64]. Microbial attack and the oxidative state of plants mediate the activation of the plant protection mechanisms against stress through different signaling pathways, which conduct the production of various protein and non-protein compounds with roles in protection [65], such as salicylic acid (2-hydroxyhippuric acid), which has also been identified in our samples through LC-MS; salicylic acid levels are known to increase during different type of infections (viruses, fungi, insects, bacteria), while exogenous treatment with salicylic acid improves the protection system of the host [66].

Bananas and plantains are the most commonly consumed as food and are used in medicine around the world, being an attractive source of bioactive compounds. These compounds with desirable biological properties for humans are also implicated in the plant protection strategy against pathogens, as in the case of phenolics, which are a key aspect in *Musa* spp. in the protection mechanism, as it has been found in the present study. A higher content of phenols, flavonoids, and antioxidant capacity was noted in the late stages of the infected samples compared to healthy samples, which showed a lower amount of phenolics and activity.

Identification of the phenolic compounds demonstrated the presence of relevant flavonoids that are involved in the defense mechanism of plants and which are known for their antibacterial activity related to infection by Gram-negative bacteria like *R. solanacearum* responsible for the Moko disease affecting the *Musa* spp. genus.

## 5. Conclusions

Bananas and plantains belonging to the genus *Musa* are largely consumed all over the world as food staples and for medicinal purposes, being an interesting source of bioactive secondary metabolites. These compounds, with valuable biological properties for humans as antioxidants, also play a pivotal role in the plant’s defense mechanism against pathogens. For example, phenolic compounds are essential to the defense strategy of *Musa* spp., as demonstrated in the current study, enhancing the antioxidant profile of these plants. Infected samples showcased a higher concentration of phenols, flavonoids, and antioxidant activity compared to healthy ones, which exhibited lower levels of phenolics and activity. The analysis of the phenolic compounds revealed the presence of significant flavonoids that participate in plant defense mechanisms. The flavonoids, including kaempferol, quercetin, and their glycosides, which we found in the banana samples, have antibacterial properties implicated in the fight against various pathogenic bacteria by interfering with their growth and survival.

## Figures and Tables

**Table 1:** High-resolution LC-MS analysis of the metabolites identified in *Musa cavendish* at Stage 1 of Moko disease.

HPLC-MS-NEGATIVE IONS						
ID	Proposed Compound Identity	Molecular Formula	Retention Time	Molecular Ion	Plant Organ	Previously Found in *Musa* spp.
8	Decanoic acid	C_10_H_20_O_2_	1.17	M-H	Both	[35]
25	8-hydroxy-2,7,7,11,15-pentamethyl-5,12,16-trioxapentacyclo [9.8.0]nonadec-13(18)-ene-3,17-dione	C_25_H_40_O_6_	1.15	M-H	Both	
37	Caffeyl alcohol	C_9_H_10_O_3_	1.15	M-H	Both	
77	Sucrose	C_12_H_22_O_11_	1.19	M-H	Both	
92	Citric acid	C_6_H_8_O_7_	1.18	M-H	Both	[35]
94	N-Benzoyl-D5-glycine	C_7_H_4_D_5_NO_3_	1.21	M-H	Both	
98	α, α-Trehalose	C_12_H_22_O_11_	1.21	M-H	Both	
102	Shikimic acid	C_7_H_10_O_5_	1.20	M-H	Both	[35]
124	Glucose, Fructose, Mannose, Galactose	C_6_H_12_O_6_	1.23	M-H	Both	[36]
131	Isoorientin	C_21_H_20_O_11_	1.30	M-H	Both	
187	Kaempferol 7-neohesperidoside	C_27_H_30_O_15_	1.56	M-H	Both	[37]
188	Coumaroyl + C_6_H_9_O_8_	C_15_H_12_O_8_	1.62	M-H	Both	
192	Isorhamnetin 3-rutinoside	C_27_H_30_O_15_	1.59	M-H	Both	[38]
198	Trihydroxyflavone C-hexoside C-pentoside	C_23_H_46_O_7_	1.56	M-H	Both	
211	Geniposide	C_17_H_24_O_10_	1.59	M+HCOO	Both	
213	Rutin	C_27_H_30_O_16_	1.62	M-H	Both	[39]
215	2-Isopropylmalic acid	C_7_H_12_O_4_	1.59	M-H	Both	[36]
220	Epicatechin	C_15_H_14_O_6_	1.46	M-H	Both	[40]
251	Isovitexin (4)	C_21_H_20_O_10_	1.60	M-H	Both	
252	Spiraeoside, Spiraein, quercetin-4′-glucoside	C_21_H_20_O_12_	1.74	M+H	Both	
256	Kaempferol-3-O-glucoside	C_21_H_20_O_11_	1.80	M-H	Both	
259	Isoorientin	C_19_H_18_O_10_	13.48	M-H	Leaf	
268	Pentose-Hexose + C_10_H_17_	C_15_H_22_O_10_	18.59	M+HCOO	Both	
313	(10E,15E)-9,12,13-trihydroxyoctadeca-10,15-dienoic acid	C_18_H_32_O_5_	21.48	M-H	Both	
322	Labetalol, 2-hydroxy-5-[1-hydroxy-2-(4-phenylbutan-2-ylamino) ethyl] benzamide	C_19_H_25_N_3_O_2_	23.23	M-H	Both	
331	Dienogest, 2-[(8S,13S,14S,17R)-17-hydroxy-13-methyl-3-oxo-1,2,6,7,8,11,12,14,15,16-decahydrocyclopenta[a]phenanthren-17-yl] acetonitrile	C_20_H_25_NO_2_	24.00	M-H	Both	
337	13-HpOTrE, 13S-hydroperoxy-9Z,11E,15Z-octadecatrienoic acid, (9Z,11E,15Z)-13-Hydroperoxy-9,11,15-octadecatrienoic acid	C_18_H_32_O_3_	24.62	M-H	Both	
338	Gibberellate, Gibberellic acid, Gibberellin A3, Gibberellin	C_19_H_22_O_6_	24.46	M-H	Both	
349	N-Acetylneuraminic acid	C_11_H_17_NO_8_	25.25	M-H	Both	
351	3-[4-methyl-1-(2-methylpropanoyl)-3-oxocyclohexyl] butanoic acid	C_13_H_22_O_3_	25.66	M-H	Both	
357	9-Hydroperoxy-10E,12Z-octadecadienoic acid	C_20_H_36_O_3_	26.09	M-H	Both	
364	9-hydroxy-7-(2-hydroxypropan-2-yl)-1,4a-dimethyl-2,3,4,9,10,10a-hexahydrophenanthrene-1-carboxylic acid	C_20_H_30_O_4_	26.50	M+H	Both	
384	2-Hydroxyhippuric acid	C_9_H_9_NO_4_	28.27	M-H	Both	
390	Canrenone, (9S,14S)-10,13-dimethylspiro [2,8,9,11,12,14,15,16-octahydro-1H-cyclopenta[a]phenanthrene-17,5′-oxolane]-2′,3-dione	C_22_H_28_O_3_	28.62	M-H	Both	
392	Ajmaline	C_20_H_26_N_2_O	28.55	M-H	Both	
398	Docosanol	C_22_H_46_O	28.85	M+H	Both	[41]
414	Diacylglycerol 18:3	C_39_H_68_O_5_	29.23	M+HCOO	Both	
421	Medroxyprogesterone	C_24_H_34_O_4_	29.26	M-H	Both	
422	Avocadyne Acetate	C_21_H_36_O_3_	29.51	M+H	Both	
456	7-O-Methylrosmanol	C_21_H_36_O_3_	29.90	M+H	Both	
457	Hydroxylated linoleic acid	C_18_H_32_O_4_	29.93	M-H	Both	
458	Dodecylbenzenesulfonic acid	C_18_H_30_O_3_S	30.02	M+H	Both	
466	Fucosyltransferase V	-	30.31	M+H	Both	
470	Furosemide, 4-chloro-2-(furan-2-ylmethylamino)-5-sulfamoylbenzoic acid	C_12_H_11_ClN_2_O_5_S	30.22	M-H	Both	
472	Lysophosphatidylcholine 18:3	C_27_H_50_NO_7_P	30.29	M+HCOO	Both	
474	1-[2-methyl-6-[(2S,3R,4S,5S,6R)-3,4,5-trihydroxy-6-(hydroxymethyl) oxan-2-yl] oxyphenyl] ethanone	C_14_H_20_O_8_	30.42	M-H	Both	
495	Phosphatidylinositol 16:0	C_37_H_71_O_8_P	30.67	M-H	Both	
520	[(4E)-7-acetyloxy-6-hydroxy-2-methyl-10-oxo-2,3,6,7,8,9-hexahydrooxecin-3-yl] (E)-but-2-enoate	C_14_H_20_O_7_	30.87	M+H	Both	
545	Threo-7′-O-Butylresveptero acyclic dimer	C_32_H_38_O_4_	31.19	M-H	Both	
546	Lysophosphatidylethanolamine 18:2	C_40_H_80_NO_8_P	31.08	M-H	Both	
563	Monogalactosyldiacylglycerol 18:3	C_57_H_102_O_10_P	31.24	M+HCOO	Both	
581	1-(9Z,12Z-Octadecadienoyl)-2-hydroxy-sn-glycero-3-phosphoethanolamine	C_42_H_78_NO_8_P	31.56	M-H	Both	
588	Ethylenediaminetetraacetic acid	C_10_H_16_N_2_O_8_	31.66	M-H	Both	
620	[5-acetyloxy-3-(hydroxymethyl)-2-oxo-6-propan-2-ylcyclohex-3-en-1-yl] 3-methylpentanoate	C_17_H_26_O_6_	32.15	M+H	Both	
621	Naringenin-7-O-glucoside	C_21_H_22_O_10_	32.03	M-H	Both	[39]
633	Hydroxyoctadecadienoic acid	C_18_H_32_O_3_	32.23	M-H1	Both	
661	N-(hexadecanoyl)-1-hydroxyethane-2-amide	C_20_H_41_NO_8_	32.76	M-H	Both	
684	9-Keto-octadecadienoic acid	C_18_H_3_O_3_	33.18	M-H	Both	
709	Threo-7′-O-Isopropylresvepterol acyclic dimer	C_29_H_32_O_6_	33.87	M-H	Both	
716	Sesamin	C_20_H_18_O_6_	33.96	M+H	Both	
721	Methyl (4aR)-5,6-dihydroxy-1,1-dimethyl-7-isopropyl-2,3,4,9,10,10a-hexahydrophenanthrenanthrene-4a-carboxylate	C_23_H_32_O_4_	34.19	M+Na	Both	
733	1-Acyl-sn-glycero-3-phosphocholine	C_26_H_52_NO_7_P	34.55	M+HCOO	Both	
742	Thymol-beta-D-glucoside	C_16_H_24_O_6_	34.68	M+H	Both	
777	Beta-alanyl-L-histidine	C_9_H_14_N_4_O_3_	40.92	M+H	Both	
807	2,6-Dihydroxybenzoic acid	C_7_H_6_O_4_	41.56	M-H	Both	
822	Cystine	C_6_H_12_N_2_O_4_S_2_	42.03	M+H	Both	
824	Norethindrone	C_20_H_26_O_2_	42.07	M-H	Both	
839	Scutellarein 4′-methyl ether	C_16_H_12_O_7_	42.95	M+H	Both	
**HPLC-MS-POSITIVE IONS**						
505	Candesartan	C_24_H_20_N_6_O_3_	1.41	M+H		
568	Adenosine	C_10_H_13_N_5_O_4_	1.42	M+		
577	Ceramide	C_34_H_66_NO_3_	1.40	M+H		
722	Isoshaftoside	C_21_H_20_O_11_	1.53	-		
728	9-Methoxycamptothecin	C_22_H_20_N_2_O_5_	1.51	-		
752	Kaempferol-3-O-rutinoside	C_27_H_30_O_15_	1.57	M+H		[42]
758	Glycochenodeoxycholic acid	C_26_H_43_NO_6_	1.57	M+H		
763	Isovitexin	C_21_H_20_O_10_	1.57	-		
769	Glycolithocholic acid	C_26_H_43_NO_4_	1.61	M+H		
974	Spiraeoside	C_21_H_20_O_11_	10.98	M+H		
980	Kaempferol-7-O-neohesperidoside	C_27_H_30_O_15_	11.33	M+H		
984	Selenomethionine	C_5_H_11_NO_2_Se	11.48	M+H		
985	Phytol	C_20_H_40_O	11.60	M+H		[40]
986	Orientin	C_27_H1_20_O_11_	11.67	M+H		
990	Cis-Nerolidol	C_15_H_26_O	12.05	M+		
991	Luteolin 4′-O-glucoside	C_21_H_20_O_11_	12.11	M-2H		
1004	Vitexin-2′‘-O-rhamnoside	C_27_H_30_O_14_	12.67	M+H		
1012	Apigenin 7-O-neohesperidoside	C_27_H_30_O_15_	12.91	M+H		
1018	5,7-dihydroxy-2-(4-hydroxy-3-methoxyphenyl)-3-[3,4,5-trihydroxy-6-[[(2R,3R,4R,5R,6S)-3,4,5-trihydroxy-6-methyloxan-2-yl] oxymethyl] oxan-2-yl] oxychromen-4-one	C_27_H_30_O_17_	13.21	M+H		
1731	6-Acetoxy-9-benzoyloxy-1,8-dihydroxydihydro-β-agarofuran	C_26_H_30_O_8_	28.93	M+H		
1741	Methyl 4-hydroxy-3,5-dimethoxybenzoate	C_10_H_12_O_5_	29.02	-		
1755	Lauryl diethanolamide	C_14_H_31_NO_2_	29.19	-		
1765	(Z)-9,12,13-trihydroxyoctadec-15-enoic acid	C_18_H_34_O_5_	29.24	M-H		
1788	(1R,9S,10S)-3,4-dihydroxy-11,11-dimethyl-5-(propan-2-yl)-16-oxatetracyclo [7.5.2.0] hexadeca-2(7),3,5-triene-8,15-dione	C_19_H_22_O_5_	29.26	M-H		
2542	Dibutylphthalate	C_16_H_22_O_4_	33.90	M+H		
2543	1-Palmitoylglycerophosphocholine	C_24_H_50_NO_7_P	33.88	M+		
2544	Diacylglycerol trimethylhomoserine	C_31_H_60_NOPS	33.92	M+H		
2548	1-Hexacosanol	C_26_H_54_O	33.94	-		
2551	1-Oleoylglycerophosphocholine	C_30_H_60_NO_7_P	34.00	M+		
2614	Beta-Peltatin	C_18_H_23_NO_5_	34.46	M-H		
2624	8-(2-hydroxy-3-methoxy-3-methylbutyl)-7-methoxychromen-2-one	C_16_H_18_O_5_	34.56	M+NH4		

## Data Availability

The original contributions presented in the study are included in the article, further inquiries can be directed to the corresponding author due to privacy.
